# Tooth loss and obstructive sleep apnoea

**DOI:** 10.1186/1465-9921-7-8

**Published:** 2006-01-17

**Authors:** Caterina Bucca, Alessandro Cicolin, Luisa Brussino, Andrea Arienti, Alessandra Graziano, Francesco Erovigni, Paolo Pera, Valerio Gai, Roberto Mutani, Giulio Preti, Giovanni Rolla, Stefano Carossa

**Affiliations:** 1 Department of Biomedical Sciences and Human Oncology, University of Turin, Italy; 2 Sleep Medicine Center, Department of Neurosciences, University of Turin, Italy and IRCCS Ist. Auxologico Italiano; 3 S. Giovanni Battista Hospital, Turin, Italy

## Abstract

**Background:**

Complete tooth loss (edentulism) produces anatomical changes that may impair upper airway size and function. The aim of this study was to evaluate whether edentulism favours the occurrence of obstructive sleep apnoea (OSA).

**Methods:**

Polysomnography was performed in 48 edentulous subjects on two consecutive nights, one slept with and the other without dentures. Upper airway size was assessed by cephalometry and by recording forced mid-inspiratory airflow rate (FIF_50_). Exhaled nitric oxide (eNO) and oral NO (oNO), were measured as markers of airway and oropharyngeal inflammation.

**Results:**

The apnoea/hypopnoea index (AHI) without dentures was significantly higher than with dentures (17·4 ± 3·6 versus 11·0 ± 2·3. p = 0·002), and was inversely related to FIF_50 _(p = 0·017) and directly related to eNO (p = 0·042). Sleeping with dentures, 23 subjects (48%) had an AHI over 5, consistent with OSA, but sleeping without dentures the number of subjects with abnormal AHI rose to 34 (71%). At cephalometry, removing dentures produced a significant decrease in retropharyngeal space (from 1·522 ± 0·33 cm to 1·27 ± 0·42 cm, p = 0·006). Both morning eNO and oNO were higher after the night slept without dentures (eNO 46·1 ± 8·2 ppb versus 33·7 ± 6·3 ppb, p = 0·035, oNO 84·6 ± 13·7 ppb versus 59·2 ± 17·4 ppb, p = 0·001).

**Conclusion:**

These findings suggest that complete tooth loss favours upper airway obstruction during sleep. This untoward effect seems to be due to decrease in retropharyngeal space and is associated with increased oral and exhaled NO concentration.

## Background

Increased pharyngeal collapsibility is a common cause of obstructive sleep apnoea (OSA). It results from the combination of anatomical abnormalities of the upper airway with changes in neural activation mechanisms [1,2]. Several structural changes in facial morphology have been implied in OSA pathogenesis, such as retrognatic mandibles, posteriorly placed pharyngeal walls and large tongues and soft palate [3]. Although the absence of teeth produces prominent anatomical changes that may influence upper airway size and function, such as loss of the vertical dimension of occlusion, reduction of the lower face height and mandible rotation [4], its role in the pathogenesis of OSA has not been investigated. We previously observed an OSA patient who, after extraction of all his teeth, developed marked worsening of his cardiorespiratory status, associated with doubling of the number of apnoea/hypopnoea episodes per hour (apnoea/hypopnoea index = AHI) [5]. A similar increase in AHI was observed in other six edentulous OSA patients when sleeping without dentures, suggesting that edentulism may favour upper airway collapse during sleep. In this regard, we previously observed that in edentulous subjects, recording lung function tests without dentures, produces mild but significant decrease in inspiratory airflow rates [6], that are known to reflect upper airway patency [7]. Another factor contributing to upper airway narrowing may be pharyngeal inflammation due to dentures wearing [8] that might amplify the inflammation produced by repeated airway closure and reopening characteristics of OSA [9,10].

The aims of the present study were to assess whether edentulism favours the occurrence of obstructive sleep apnoea, and if this untoward effect is related to changes in upper airway size, and to oral and airway inflammation. To this purpose, 48 edentulous subjects wearing complete dentures underwent polysomnography on two consecutive nights, one slept with and the other slept without dentures. Upper airway size was assessed by cephalometry and by recording inspiratory airflow rates, inflammation was evaluated by measuring oral and exhaled NO.

## Methods

### Patients

All the edentulous outpatients wearing complete dentures, who attended the Internal Medicine Clinic and the Sleep Medicine Center in the period 1999 to 2003, were invited to participate in the study. The selection criteria were ability to give sufficient collaboration to the tests, stable clinical conditions, absence of severe cardiologic, neurologic, and respiratory diseases and of recent acute upper or lower airway infections. The subjects were informed about the aims and the modality of the study, which implied two consecutive full-night polysomnography (PSG) recordings, performed either in hospital (attended PSG) or using a portable instrument (ambulatory PSG). Out of the 64 subjects selected, 16 refused, and 48 gave their written consent to participate in the study: 28 accepted ambulatory PSG and 20 accepted attended PSG.

### Procedures

The protocol of the study, approved by the local ethics committee, included clinical examination, lung function tests, arterial blood gas analysis, measurement of exhaled nitric oxide (eNO) level, and PSG with and without dentures, in randomized order. In the subjects who accepted attended PSG, eNO and oral NO (oNO) were measured at awakening after sleeping with and without dentures and cephalometry was assessed with and without dentures.

#### Lung function tests

Lung volumes and flow-volume loop were measured using the computerized spirometer Baires (Biomedin, Padua, Italy), and were expressed as percent of predicted [11]. Forced mid-inspiratory flow (FIF_50_) was used as the arbitrary index of upper aiway patency [7], as we previously found that it is mostly influenced by removing dentures [6]. Predicted values for FIF_50 _were obtained in our laboratory, from a population of 75 asymptomatic healthy subjects without OSA, matched for age and sex.

Arterial blood gases were measured using ABL 330 analyser (Radiometer, Copenhagen, Denmark)

#### Polysomnography

Overnight PSG was performed using the Aurora system for in-laboratory recording and the H2O system for ambulatory recording (Grass-Telefactor, ASTRO-Med Inc., RI, USA) according to the guidelines of the American Academy of Sleep Medicine (ASDA) [12], using two EEG channels (C3-A2, O1-A2), two electro-oculogram channels (EOG), surface mentalis electromyography (EMG), bilateral surface anterioris tibialis EMG, electrocardiography (modified lead II) (ECG), oro-nasal air flow (2 channel thermistor), respiratory movements (thoracic and abdominal belts), snoring and body position; oxygen saturation (SaO_2_). The ASDA criteria were used for staging sleep and for apnoea/hypopnoea definition [13]. OSA was diagnosed when the AHI was over 5.

#### Exhaled and oral NO level

Both eNO and oNO were measured using a chemiluminescence analyser (SIEVERS 280 NOA, Boulder, CO). eNO was measured off-line according to American Thoracic Society/European Respiratory Society recommendations [14]. Exhaled air was collected using a pressure-regulated flow-restricted apparatus (Sievers, Boulder, CO); the measure was performed within 4 hours from collection.

In subjects who underwent attended PSG, oNO and eNO were measured at the awakening after the night slept with and after that slept without dentures. Oral NO was measured using a special device, as previously described [15], 15 minutes after mouth rinsing with water. The device consists of a holed metal tube, welded at right angle to a shaped plate which presses the tongue against the soft palate so as to isolate the mouth from upper and lower airway and from gastric cavity. A soft mouthpiece, fitted on the tube, maintains the mouth closed off and fastens device position. A two-way valve connects the plate to a glass syringe, for delivery and withdrawal of air samples to and from the oral cavity, and, through a wide-bore Teflon tube (2 mm internal diameter), to the NO meter. Thirty ml NO-free air (NO below 5 ppb) are injected with a glass syringe through the device into the closed empty mouth and maintained for 30 seconds; thereafter, the air is withdrawn and shifted to the NO analyser.

#### Cephalometry

The radiographs were taken at 2.5 meters distance, in supine relaxed position, to simulate, as much as possible, the night-time position. The patient's head was stabilized to minimize the risk of unintentional movement. Two radiographs were taken, one without and one with dentures. The radiographs were performed with a digital system of image acquisition (Planmeca 2002 cc Proline, OWAV, ASENTAJANKATU 6008109, HELSINKY, FINLAND), that allows to obtain a precise contour of the soft structures. The retropharyngeal space was measured in centimetres at three levels as suggested by Athanasiou et al [16], as the mean of the three measures. The narrowest distance from the tongue base to the posterior pharyngeal wall (PAS) was also calculated: a value equal or below 10 mm was considered indicative of macroglossia [17]

### Statistic analysis

Data were analysed using the SAS statistical package [18]. Descriptive statistic was used to evaluate the characteristics of the study population. Student's t test for paired samples and one-way analysis of variance (ANOVA) were used to compare sleep monitoring data obtained in the night slept with dentures with those in the night without dentures. Linear simple and multiple regression analysis were used to evaluate the relationship of AHI and its variation with and without dentures, with the variables considered. The characteristics of the two groups of patients were compared using the t-test for unpaired data and the χ square test.

For each test a p value below 0,05 was considered statistically significant.

## Results

The general characteristics of the 48 subjects who completed the study are reported in Table 1.

**Table 1:** General characteristics of the 48 subjects

Age (years) Mean ± SEM	69 ± 9
BMI Mean ± SEM	27.5 ± 0.7
BMI ≥ 28 n. (%)	18 (37.5)
Men n. (%)	29 (60.4)
Current smokers n. (%)	7 (14.6)
Ex-smokers n. (%)	23 (47)
Sleeping with dentures n. (%)	30 (63)
Systemic hypertension n. (%)	19 (38)
COPD n. (%)	11 (23)
PaO_2 _below 70 mmHg n. (%)	14 (29)
FIF_50 _below 60% predicted n. (%)	32 (67)

Most of the subject were over sixty, male sex, overweight, with decreased upper airway patency (FIF_50 _< 60% predicted). The prevalence of systemic arterial hypertension [19] and of chronic obstructive pulmonary disease (COPD) [20] was that expected in the general population of same age-range.

The results of PSG showed that the apnoeic episodes were mainly obstructive in type, central sleep apnoeas representing no more than 4% of the total respiratory events, both with and without dentures. As shown in Table 2, the AHI was significantly higher and the mean SaO_2 _significantly lower in the night slept without dentures. Sleeping with dentures, the PSG tracing was consistent with OSA [13] in 23 (48%) subjects, but sleeping without dentures, the number of subjects with OSA rose to 34 (71%). In fact, the AHI increased over 5 in 11 subjects (44%) who had normal PSG sleeping with dentures.

**Table 2:** Comparison between polysomnography data in the night slept with dentures and in that slept without dentures. The values are expressed as mean ± standard error of the mean (SEM)

	Dentures on	Dentures off	P
AHI N	11.0 ± 2.3	17.4 ± 3.6	0.002
Mean SaO_2_%	93.2 ± 0.4	92.9 ± 0.5	0.006
Nadir SaO_2_%	83.9 ± 0.9	82.1 ± 1.1	0.091

Interestingly, the AHI without dentures was significantly inversely related to FIF_50_% (r = 0.371, p = 0.017) and directly related to eNO (r = 0.317, p = 0.042); FIF_50_% and eNO were also significantly related each other (r = 0.359, p = 0.024). The other lung function tests, besides FIF_50_%, as well as age, sex, BMI, smoking history, COPD, PaO_2_, PaCO_2_, showed no significant influence on the AHI values, either with or without dentures. For these reasons, none of the variables entered the model of multiple regression analysis.

The findings in the 20 subjects undergone attended PSG, showed that both mean retropharyngeal space and PAS were significantly decreased by removing dentures. The PAS with dentures was consistent with macroglossia (below 10 mm) [17] in 16 of the 20 subjects. Morning eNO and oNO were significantly higher after the night slept without dentures than after that with dentures, see Table 3.

**Table 3:** Supine cephalometry values obtained with and without dentures, and morning values of oral and exhaled NO after sleeping with and without dentures in the 20 subjects who underwent assisted polysomnography

	With dentures	Without dentures	P
*Cephalometry*			
Mean retropharyngeal space mm	15.2 ± 3.3	12.7 ± 4.2	0.0006
PAS mm	6.9 ± 1.0	4.8 ± 0.8	0.035

*Morning NO*			
Oral NO ppb	59.2 ± 17.4	84.6 ± 13.7	0.001
Exhaled NO ppb	33.7 ± 6.3	46.1 ± 8.2	0.035

## Discussion

The results of this study demonstrate that edentulism favors upper airway obstruction during sleep. In fact, both AHI and mean SaO_2 _were significantly worse in the night slept without dentures than in the night slept with dentures, see Table 2. The decrease in pharyngeal airway by edentulism is supported by the findings of cephalometry, showing that the retropharyngeal space was significantly decreased by removing dentures. The close inverse relationship of AHI without dentures with inspiratory airflow rates (FIF_50_) that are known to reflect upper airway patency [6] supports the hypothesis that edentulism-induced decrease in pharyngeal caliber influences OSA. Present knowledge suggests that edentulism acts through complex mechanisms, ranging from simple anatomical changes to impairment of neural reflexes and neuromuscular activity. The main anatomical changes caused by the loss of natural teeth consist in loss of the vertical dimension of occlusion, reduction of the lower face height and rotation of the mandible, all leading to occlusion disharmony [3]. Moreover, long lasting edentulism causes abnormal tongue position [21], impairs tongue motor skill [22] and favors macroglossia, as the tongue fills the space of missing natural teeth and may thus predispose to retrolingual space obstruction, that is very common in OSA patients [23]. Actually, on cephalometry, the PAS was consistent with macroglossia [17] in most of our subjects.

Edentulism-induced neuromuscular dysfunction is supported by the observations that tooth loss is associated with decreased oral innervation [24], loss in oro-pharyngeal coordination and impaired osseoperception [25], that are not re-established by complete denture restoration. These abnormalities may favor upper airway collapse by impairing the activation of the pharyngeal dilators in response to upper airway stimuli [26].

The close relationship among eNO, the marker of airway inflammation [27], with both FIF_50 _and AHI without dentures, suggests that inflammation contributes to upper airway narrowing. The finding that both morning eNO and oNO were significantly higher after the night slept without dentures, during which there were more obstructive episodes, supports the concept that in OSA repeated episodes of airway closure and reopening causes airway inflammation [10,11]. Soft palate and uvula inflammation have been demonstrated in patients with OSA [10,28] and in edentulous subjects such inflammation might be amplified by the mucosa irritation induced by dentures wearing [8,29]. Actually, the posterior ridge of the denture is designed to fit on the border between the hard and soft palate and this boundary area is rich of nerve fibers and contains several inflammatory cytokines [30] that might be released in response to dentures pressure, giving rise to inflammatory edema.

Interestingly, the effect of removing dentures during sleep on breathing, was more evident in subjects who had normal PSG sleeping with dentures. Actually, in about half of them (44%) the AHI rose over 5, the cutoff to define OSA, sleeping without dentures, indicating that in these subjects edentulism might be the only cause of OSA.

## Conclusion

In conclusion, the findings of this study indicate that edentulism may worsen OSA, particularly in subjects who have no respiratory disturbances sleeping with dentures. Edentulism might act by modifying anatomy and function of the pharyngeal airway and of tongue and by favouring inflammatory edema.

Epidemiological studies estimate that edentulism troubles about 18 % of patients older than 60 years and that such prevalence will remain constant over the next 30 years [31]. Considering the high prevalence of OSA in the advanced age, it is conceivable that a consistent number of elderly people is at risk of edentulism-induced worsening of OSA and, consequently, of OSA related morbidity and mortality. Dentists generally recommend to remove dentures during the night, to limit the risk of denture irritations [29]. Our findings suggest that the advantages of removing dentures during sleep should be weighted against the risk of favouring upper airway collapse.

## List of abbreviations used

AHI : number of episodes of apnoea/hypopnoea per hour

COPD : chronic obstructive pulmonary disease

eNO : exhaled nitric oxide

FIF_50 _: forced mid-inspiratory flow

oNO : oral nitric oxide

OSA : obstructive sleep apnoea

PAS : posterior airway space

PSG : polysomnography

SaO2 : oxyhemoglobin saturation

## Competing interests

The author(s) declare that they have no competing interests.

## Authors' contributions

*Study concept and design: *BC, CS, CA.

*Acquisition of data: *BC, CA, AA, EF, BL, GA, GV.

*Analysis and interpretation of data: *BC, CA, GA,

*Drafting of the manuscript: *BC, CA, RG.

*Critical revision of the manuscript for important intellectual content: *MR, PG, RG, GV, PP

*Statistical analysis: *BC, CA.

*Obtained funding: *CS.

*Administrative, technical, or material support: *BC, CA, GA, AA

*Study supervision: *MR, PG, RG, GV

## Sponsor details

This paper has been funded by the Italian Ministry of Instruction, University and Research (MIUR): prot. 2002065529
